# Association of Vitamin D in Different Trimester with Hemoglobin during Pregnancy

**DOI:** 10.3390/nu14122455

**Published:** 2022-06-14

**Authors:** Shuting Si, Zhicheng Peng, Haoyue Cheng, Yan Zhuang, Peihan Chi, Xialidan Alifu, Haibo Zhou, Minjia Mo, Yunxian Yu

**Affiliations:** 1 Department of Public Health, and Department of Anesthesiology, Second Affiliated Hospital of Zhejiang University School of Medicine, Hangzhou 310058, China; 21818499@zju.edu.cn (S.S.); 22018678@zju.edu.cn (Z.P.); 3150101365@zju.edu.cn (H.C.); yanzhuang@zju.edu.cn (Y.Z.); 22118872@zju.edu.cn (P.C.); 3130100017@zju.edu.cn (X.A.); 11918158@zju.edu.cn (H.Z.); minjiamo@zju.edu.cn (M.M.); 2 Department of Epidemiology & Health Statistics, School of Public Health, School of Medicine, Zhejiang University, Hangzhou 310058, China

**Keywords:** hemoglobin, vitamin D, iron supplementary, pregnancy

## Abstract

The association between vitamin D and hemoglobin has been suggested. Vitamin D can affect erythropoiesis by the induction of erythroid progenitor cell proliferation and enhance iron absorption by regulating the iron-hepcidin-ferroportin axis in monocytes. However, this relationship in pregnant women is scarce. The purpose of this study was to investigate the association between plasma vitamin D levels with hemoglobin concentration in pregnant women considering each trimester and iron supplementation. The data were obtained from Zhoushan Pregnant Women Cohort, collected from 2011 to 2018. Plasma 25(OH)D was measured in each trimester using liquid chromatography–tandem mass spectrometry. Generalized estimating equations and multiple linear regressions were performed. Finally, 2962 pregnant women and 4419 observations in the first trimester were included in this study. Plasma 25(OH)D in first trimester (T1) (β = 0.06, *p* = 0.0177), second trimester (T2) (β = 0.15, *p* < 0.0001), and third trimester (T3) (β = 0.12, *p* = 0.0006) were positively associated with Hb. Association between plasma 25(OH)D levels in T1 and Hb concentration was positively associated with gestational age (β = 0.005, *p* = 0.0421). Pregnant women with VD deficiency in T1 (OR = 1.42, 95% CI: 1.07–1.88) or T2 (OR = 1.94, 95% CI: 1.30–2.89) presented an increased risk of anemia, compared with women without VD deficiency. Moreover, the significant relationship between VD and Hb was only observed among women with iron supplementation during pregnancy. Plasma 25(OH)D levels in each trimester were positively associated with Hb concentration. Iron supplementation might be an important factor affecting the relationship between VD and Hb.

## 1. Introduction

Anemia is a serious global public health problem particularly affecting young children and pregnant women. In 2019, the global prevalence of anemia among pregnant women aged 15–49 years was 36% (95% CI: 34–9%) and it was 27% (95% CI: 21–35%) in east and southeast Asia [1]. In China, the reported prevalence was relatively low, varying from 10.5% to 23.5% [2,3,4,5], but it still cannot be ignored. Because the adverse pregnancy outcomes (such as gestational diabetes, polyhydramnios, preterm birth, low birth weight, neonatal complications, and NICU admission) were significantly higher among pregnant women with anemia [2,6,7]. The most common cause of anemia is poor nutrition, especially iron deficiency. In recent years, it has been suggested that vitamin D (VD) deficiency may also be a key factor. VD insufficiency or deficiency was also prevalent in pregnant women around the world. The prevalence among Asian pregnant women was 45~98% [8], and it was about 96.0% among Chinese pregnant women [9]. As Ellen et al. summarized, epidemiological studies have linked VD deficiency to an increased risk of anemia in various healthy and diseased populations [10,11,12,13]. In terms of biological mechanisms, VD affects hemoglobin concentration by regulating erythropoiesis, immune cells, and hepcidin production [14,15,16,17]. However, evidence for such an association in pregnant women remains scarce and contradictory. In 2022, one meta-analysis including eight observational studies concluded that VD deficiency might be a risk factor for anemia in pregnancy [18]. However, most studies included in this meta-analysis had moderate or low methodological quality and a limited sample size. Moreover, most studies measured 25(OH)D and hemoglobin (Hb) only once during pregnancy without considering changes in the level of 25(OH)D and Hb throughout pregnancy. Some studies even did not report which trimester or gestational age was to measure 25(OH)D and Hb. In addition, one study was conducted on adolescent pregnant women which might not be able to be extrapolated to adult pregnant women.

Therefore, in this cohort study, we examined plasma 25(OH)D levels across all the three trimesters and extracted each Hb from each prenatal visit to further explore the association of VD in different trimesters as well as the dynamic change of VD with Hb during pregnancy. Meanwhile, whether iron supplementary could influence the association of VD with Hb was explored.

## 2. Materials and Methods

### 2.1. Study Design and Participants

This was a prospective cohort study based on the Zhoushan Pregnant Women Cohort (ZPWC) from August 2011 to May 2018. ZPWC is an ongoing prospective cohort conducted in Zhoushan Maternal and Child Health Care Hospital, Zhejiang. Pregnant women were recruited at their first prenatal visit between 8 and 14 gestational weeks. The inclusion and exclusion criteria have been described previously [19]. Briefly, pregnant women aged between 18 and 45 years without serious physical, or mental health disease, threatened abortion or dysontogenesis, and who have conducted plasma 25(OH)D and Hb measurement in T1 and at least two times Hb measurement during pregnancy were included in this study. Before participating in this study, written informed consents were obtained from all pregnant women. The study protocol was approved by the ethics board of Zhejiang University School of Medicine.

### 2.2. Date and Blood Sample Collection

Pregnant women enrolled in the ZPWC were interviewed with a structured questionnaire face-to-face by a well-trained interviewer to collect information on socio-demographic characteristics, lifestyle, and health behavior in the first trimester (T1: 8th to 13th gestational week). Participants would be followed up in the second trimester (T2: 24th–27th gestational week), third trimester (T3: 32nd–36th gestational week), and 42nd day postpartum with the corresponding questionnaire. At each visit, 5 mL fasting venous blood was drawn and centrifuged at 4 °C. Plasma and white blood cells were separated and stored at −80 °C until use. The Hb concentration was measured during the routine examination, and we extracted it from the electronic medical records system. According to the frequency of blood examination during prenatal visits, Hb may be measured multiple times inT2 and T3. Therefore, we further categorized the Hb concentrations into seven periods (before 14th, 14 to 17th, 18 to 22nd, 23 to 27th, 28 to 31st, 32 to 35th, and 36 to 42nd gestational week).

### 2.3. Measurement of Plasma 25(OH)

Plasma 25(OH)D2 and 25(OH)D3 concentrations in T1, T2, and T3 were measured by Liquid chromatography–tandem mass spectrometry (API 3200MD (Applied Biosystems/MDS Sciex, Framingham, MA, USA)) and reported in ng/mL, respectively. The lowest sensitivity of the measurement was 2 ng/mL for 25(OH)D2 and 5 ng/mL for 25(OH)D3, respectively. The intra-assay coefficient variances (CVs) were 1.47–7.24% and 2.50–7.59% for 25(OH)D2 and 25(OH)D3, respectively, and the inter-assay CVs were 4.48–6.74% and 4.44–6.76% for 25(OH)D2 and 25(OH)D3, respectively [15]. The 25(OH)D concentrations were the sum of 25(OH)D2 and 25(OH)D3 concentrations.

### 2.4. Variable Definition

According to the Endocrine Society Clinical Practice Guideline, VD deficiency was defined as 25(OH)D <20 ng/mL [20]. To identify the presence of anemia during pregnancy, we used the anemia criteria from the Centers for Disease Control and Prevention criteria in the United States: <110 g/L in the first trimester, <105 g/L in the second trimester, and <110 g/dL in the third trimester [21]. Gestational age was calculated according to the date of visiting the hospital and conception date (the date of last menstruation and confirmed by B ultrasound). Pre-pregnancy BMI was calculated by dividing the weight in kilograms by the square of height in meters. Considering the duration and intensity of sunshine, we categorized seasons into two groups, summer/autumn (June to November) and winter/spring (December to May). Gravidity was categorized into 1 time, 2 times, 3 times, and ≥4 times, and parity was categorized into 0 and ≥1 times. Education was categorized into junior high school and below, high school, college, and above. Iron supplementary was categorized into two groups (never, and at least one trimester during pregnancy). The categorical variable was defined as “missing” if there was no response.

### 2.5. Statistical Analysis

Continuous variables and categorical variables were presented as mean ± SD and frequency (percentage), respectively. To compare the characteristics between groups, Student’s t-test and the Chi-square test were used for continuous variables and categorical variables, respectively. Due to longitudinal repeated measures of Hb during pregnancy, generalized estimating equations (GEE) were used to analyze the association between VD or VD deficiency in each trimester and Hb or anemia during pregnancy after the corresponding 25(OH)D measurement. We also analyzed the interaction between VD and gestational age of Hb measurement on Hb in GEE models. Considering that the iron supplementation could be a key confounder, analyses were further conducted and stratified according to whether iron was supplemented during pregnancy. In addition, the association of VD in each trimester with Hb concentrations in different gestational ages and the association between VD change and Hb change between different trimesters were analyzed using multiple linear regression. Models were adjusted for potential gestational age at Hb measurement, age, gestational age at 25(OH)D measurement, gravidity, parity, season at 25(OH)D measurement, pre-pregnancy BMI, smoking, drinking and tea before pregnancy, sleep quality and physical frequency at trimester of 25(OH)D measurement, weight gain, corresponding baseline Hb, and whether iron was supplementary or not, which were detailed in tables. In GEE models, weight gain was weight at the last Hb test subtracting weight at the corresponding VD measurement, and baseline Hb was defined as the corresponding Hb that detected the nearest VD. In linear models, weight gain was weight at the corresponding Hb test subtracting weight at the corresponding VD measurement, and baseline Hb was defined as the corresponding Hb that detected the nearest VD. The level of *p* < 0.05 was considered statistically significant for all tests in this study. All analyses were performed using R software (version 4.0.2) (http://www.R-project.org accessed on 22 June 2020).

## 3. Results

Finally, 2962 pregnant women in T1 were included in this study. Of which, 516 pregnant women with data of plasma 25(OH)D and questionnaire in T2 and 293 pregnant women with data of plasma 25(OH)D and questionnaire in T3. In terms of the observation number, there were 4419 observations in T1, 1053 observations in T2, and 337 observations in T3. The prevalence of VD deficiency in T1, T2, and T3 was 65.87%, 48.06%, and 46.42%, respectively. The prevalence of anemia in seven groups (before 14th (*n* = 2962), 14 to 17th (*n* = 1156), 18 to 22nd (*n* = 1058), 23 to 27th (*n* = 1098), 28 to 31st (*n* = 696), 32 to 35th (*n* = 658), and from 36 to 42nd (*n* = 555) gestational age) was 4.19%, 4.07%, 8.60, 12.39%, 33.33%, 34.65 and 22.70%, respectively. Pregnant women with VD deficiency in T1 had a higher proportion of first pregnancy, nulliparous, drinking before pregnancy, poor sleep quality, 25(OH)D measurement in winter or spring, iron supplementation at T1 and T2 (*p* < 0.05). Pregnant women with VD deficiency in T1 were younger and had a lower pre-pregnancy BMI, lower Hb, and higher weight gain (*p* < 0.05) (Table 1).

### 3.1. Association between Plasma 25(OH)D in Different Trimester and Hemoglobin after Corresponding 25(OH)D Measurement

Due to repeated measurements of Hb during pregnancy, we used GEE models to assess the association of plasma 25(OH)D in each trimester with Hb that was detected during pregnancy but after the corresponding 25(OH)D measurement, respectively. The level of plasma 25(OH)D in T1(β = 0.06, *p* = 0.0177), T2 (β = 0.15, *p* < 0.0001), and T3 (β = 0.12, *p* = 0.0006) was positively associated with Hb concentrations even if models were adjusted for corresponding confounders, respectively (Table 2). In addition, considering that the association might be different as the gestational age changed, we also created models with the interaction between plasma 25(OH)D in each trimester and the gestational age at the Hb measurement, respectively. Interestingly, we observed that only *p* for the interaction between plasma 25(OH)D in T1 and gestational age at Hb measurement was statistically significant and revealed that the association of plasma 25(OH)D in T1 with Hb concentrations was positively associated with gestational age (β = 0.005, *p* = 0.0421). However, no interaction was found between plasma 25(OH)D in T2 (*p* = 0.2076) or T3 (*p* = 0.2405) and the gestational age at which Hb was detected after the corresponding 25(OH)D measurement (Table 2).

Iron supplementation may be an important confounding factor. Therefore, we further conducted analyses stratified by whether iron was supplemented or not during pregnancy. As shown in Table 3, we found plasma 25(OH)D in T1 (β = 0.09, *p* = 0.0016) and T2 (β = 0.17, *p* < 0.0001) were significantly associated with Hb concentrations that were detected after 25(OH)D measurement among those who received iron supplements during pregnancy. However, the results did not show the interaction between plasma 25(OH)D and iron supplementation. The positive association of plasma 25(OH)D in T3 with Hb concentrations that were detected after the 25(OH)D measurement, and both existed regardless of whether iron supplementation occurred during pregnancy (with iron supplementation, β = 0.08, *p* = 0.0483; without iron supplementation, β = 0.15, *p* = 0.0046).

### 3.2. Association between VD Deficiency in Different Trimester and Anemia after Corresponding 25(OH)D Measurement

Considering the clinical significance, we analyzed the relationship between VD deficiency and anemia. Compared to pregnant women without VD deficiency in T1, pregnant women with VD deficiency in T1 were associated with a higher risk for anemia after the 25(OH)D measurement (OR = 1.42, 95% CI: 1.07–1.88) (Table 4). Similar results of the association between VD deficiency in T2 and anemia were revealed (OR = 1.94, 95% CI: 1.30–2.89) (Table 4). Consistent with the association between plasma 25(OH)D and Hb, we only found that VD deficiency in T1 (OR = 1.68, 95% CI: 1.20–2.34) and T2 (OR = 2.02, 95% CI: 1.27–3.22) was significantly associated with a higher risk for anemia after the 25(OH)D measurement among those who received iron supplements during pregnancy (Table 5). However, no significant association between VD deficiency in T3 and anemia was observed among the women with or without iron supplementation (Table 4 and Table 5).

### 3.3. Association between Plasma 25(OH)D in Different Trimester and Hb in Different Gestational Age after Corresponding 25(OH)D Measurement

In order to further evaluate whether the association of plasma 25(OH)D in different trimesters and Hb concentrations became stronger or not as the gestational age increased, we assessed the association between plasma 25(OH)D in different trimesters and Hb in different gestational ages, respectively. When only adjusted for gestational age at Hb measurement in model 1, the effects of VD per ng/mL in T1 on Hb of gestational age from 14th to 42nd week ranged from 0.02 to 0.17 g/L. After adjustment for corresponding confounders, the relationship that increased with gestational age became less obvious. However, in model 4, when we adjusted for the variables in model 3 but Hb in T1 was instead of baseline Hb, a similar trend to model 1 was observed (Table 6). Consistent with the results of GEE models, we failed to find the changing trend of the association between plasma 25(OH)D in T2 and Hb from 28th to 42nd gestational age or plasma 25(OH)D in T3 and Hb (Supplementary Table S1). When stratified by iron supplementation during pregnancy, the positive associations between plasma 25(OH)D in different trimesters and Hb concentrations in different gestational ages were only observed among those who received iron supplements during pregnancy (Supplementary Table S2).

### 3.4. Association between Plasma 25(OH)D Change and Hemoglobin Change between Different Trimesters

In addition, we also analyzed the association of the plasma 25(OH)D change with hemoglobin change between different trimesters to further confirm the association between plasma 25(OH)D and Hb. The results are shown in Table 7. The change of 25(OH)D from T1 to T2 had no association with the change of Hb from T1 to T2 (β = −0.00, *p* = 0.9225). However, the change of plasma 25(OH)D from T1 to T3 was positively associated with the change of Hb from T1 to T3 (β = 0.15, *p* = 0.0027), and the change of plasma 25(OH)D from T2 to T3 was positively associated with the change of Hb from T2 to T3 (β = 0.13, *p* = 0.0312).

## 4. Discussion

In the present study, the levels of plasma 25(OH)D in T1, T2, and T3 were positively associated with Hb concentrations after the 25(OH)D measurement and the association of plasma 25(OH)D in T1 and Hb concentration became stronger as the gestational age increased. Meanwhile, pregnant women with VD deficiency in T1 or T2 had an increased risk of anemia, respectively, compared with those without VD deficiency. However, the significant relationship between VD and Hb was only observed among those with iron supplementation during pregnancy.

Some previous studies have reported that pregnant women with VD deficiency had a significantly higher risk of anemia, but in their studies, only one or two measurements of 25(OH)D were conducted [22,23,24,25,26]. However, in our study, we measured 25(OH)D at three trimesters, respectively; meanwhile, we also found that 25(OH)D in T1, T2, and T3 were positively correlated with Hb, respectively. VD deficiency in T1 or T2 was associated with an increased risk of anemia. The finding, that the change of plasma 25(OH)D from T1 to T3 was positively associated with the change of Hb from T1 to T3, further increased the evidence of the relationship between VD and Hb. Thomas et al. [25] found that it could be partly explained by the mediation of erythropoietin, which was recognized in hemodialysis patients clinically [16]. Moreover, VD deficiency might also stimulate immune cells in the bone marrow microenvironment to produce cytokines, resulting in impaired red blood cell production [15]. In addition, hepcidin was also recognized as a key role in the association between VD and Hb. Increased hepcidin could inhibit enterocytes to absorb iron and lead to anemia, but VD could suppress the expression of hepcidin mRNA and enhance iron absorption by regulating the iron-hepcidin-ferroportin axis in monocytes [14,17]. However, one RCT from England reported that VD supplementation (1000 IU/day) from the first trimester had no effect on hepcidin in the third trimester [27]. There were also some studies that found no association between VD deficiency and anemia. One cross-sectional study in Sudan that enrolled 180 pregnant women found no correlation between serum 25(OH)D and Hb level (r = 0.001, *p* = 0.999) [28]. Another two cross-sectional studies in Bangladesh and Brazil used VD deficiency as an outcome and also found no association between them [10,29]. We summarized and speculated the reasons for the inconsistent results in the following aspects. First, these three studies were all cross-sectional studies with small sample sizes. Second, the relationship between VD deficiency and anemia was not the main objective of these studies and two of them took VD deficiency as the outcome in statistical models, with the latter results in the low statistical power. Third, the association between VD and anemia could be different in different trimesters. Fourth, they did not consider iron supplementation during pregnancy. In our study, it also could not be ignored that we failed to reveal the statistically significant association between VD deficiency in T3 and anemia after the VD measurement. Perhaps because of the short time interval, the effect of VD on Hb had not been fully shown. As we discovered, the association of plasma 25(OH)D in T1 and Hb became stronger as the gestational age increased. When we adjusted for potential confounders and Hb in T1, a similar trend was observed. To our knowledge, no previous study reported similar results. However, one study found a 6.97-fold increased risk of anemia at delivery in women with vitamin D deficiency [25], which was far stronger than the association between VD deficiency and anemia during pregnancy in previous studies [22,23,26] and the present study. A previous study showed that 25(OH)D3 reached a plateau in the third month in the vitamin D3 supplement group, which indicates that VD supplementation takes more than three months to have the maximal effect [30]. We also found that after adjustment for Hb in T1 (<14 gestational weeks), 25(OH)D in T1 was significantly and positively associated with Hb after 32 gestational weeks rather than that within 32 gestational weeks; furthermore, the dose-response effect of 25(OH)D in T1 and Hb measured in various gestational weeks was observed (Table 6). The time intervals between the VD measurements in T2 or T3 and Hb measurement after corresponding VD measurements were shorter than 3 months. The dose-response effect of 25(OH)D and Hb was not observed. Furthermore, for further demonstrating the dose-response effect between 25(OH)D and Hb, we evaluated the associations between 25(OH)D change and hemoglobin change between different trimesters (Table 7). We also found that the changes in VD level from T1 to T2 had a significant association with the change of Hb from T2 to T3 rather than from T1 to T2. More studies should be conducted to confirm these findings. When we further considered the influence of iron supplementation, we only found a significant correlation between VD and Hb or VD deficiency and anemia in women with iron supplementation during pregnancy. The possible reason partly resulted from the fact that VD might increase Hb by decreasing hepcidin and increasing iron absorption. When iron supplementation was present, it might provide a source for VD to promote iron absorption.

### Strengths and Limitations

Our study has some strengths. First, most previous studies focused on plasma 25(OH)D concentration and Hb concentration in a single trimester, but in this study, we evaluated the association between VD in each trimester and Hb at different gestational ages during the whole pregnancy. Meanwhile, we also explored the interaction between plasma 25(OH)D and the gestational age of Hb measurements on Hb levels in GEE models. Second, the association between the change of VD and the change of Hb was also examined to further explore the association. Third, considering the influence of iron supplementation, we also conducted a stratified analysis according to the iron supplementation variable. In addition, we also considered sleep quality, physical activity quality, and several other variables as potential confounders to improve the reliability of results. However, several limitations should be mentioned. First, ferritin, transferrin, etc., were not detected, and we could not distinguish between iron deficiency or anemia. Second, we only adjusted iron supplementation during pregnancy in the model and conducted a stratified analysis, but we did not access the frequency and amount of iron supplementation. Third, the diagnosis criteria of anemia from the Centers for Disease Control and Prevention criteria in the United States were changed across gestational trimesters, such as <110 g/L in the first trimester, <105 g/L in the second trimester, and <110 g/dL in the third trimester, and it may introduce the bias. However, other some studies have used Hb concentration below 110 g/L as the diagnosis criteria for anemia. However, Breymann [31] et al. thought that any Hb below 105 g/L could be regarded as true anemia. Fourth, parathyroid hormone and fibroblast growth factor 23 might have a damaging effect on iron metabolism and confound the association between VD and iron circulation and anemia [32], but they were not measured in our study.

## 5. Conclusions

In conclusion, the plasma 25(OH)D concentration in each trimester was positively associated with Hb concentration, and the association of plasma 25(OH)D in T1 and Hb became stronger and stronger as the gestational age increased. Iron supplementation might be an important factor affecting the relationship between VD and Hb. Both VD deficiency and anemia are very common among pregnant women. This finding indicates that VD supplementation before conception or early T1 not only improves VD deficiency but is also beneficial for the prevention of anemia, especially in pregnant women with iron supplementation. More studies are warranted to determine the association and mechanisms between VD and Hb.

## Figures and Tables

**Table 1:** The comparisons of characteristics between pregnant women with and without VD deficiency at T1.

Variables	Vitamin Deficiency at T1		*p*
	No (*n* = 1011)	Yes (*n* = 1951)	
	*n* (%)		
**Gravidity**			
1	406 (40.2)	965 (49.5)	<0.001
2	298 (29.5)	496 (25.4)	
3	176 (17.4)	252 (12.9)	
≥4	107 (10.6)	165 (8.5)	
missing	24 (2.4)	73 (3.7)	
**Parity**			<0.001
0	529 (52.3)	1165 (59.7)	
≥1	301 (29.8)	308 (15.8)	
missing	181 (17.9)	478 (24.5)	
**Education**			0.5470
Junior high school and below	90 (8.9)	154 (7.9)	
High school	198 (19.6)	364 (18.7)	
College and above	705 (69.7)	1405 (72.0)	
missing	18 (1.8)	28 (1.4)	
**Pre-pregnancy smoking**			0.0930
No	1006 (99.5)	1927 (98.8)	
Yes	4 (0.4)	23 (1.2)	
missing	1 (0.1)	1 (0.1)	
**Pre-pregnancy drinking**			0.0190
No	1003 (99.2)	1908 (97.8)	
Yes	5 (0.5)	30 (1.5)	
missing	3 (0.3)	13 (0.7)	
**Pre-pregnancy drinking tea**			0.3720
No	936 (92.6)	1790 (91.7)	
Yes	71 (7.0)	145 (7.4)	
missing	4 (0.4)	16 (0.8)	
**Physical activity frequency at T1**			0.2400
Never	859 (85.0)	1654 (84.8)	
<3 times per week	109 (10.8)	236 (12.1)	
≥3 times per week	28 (2.8)	35 (1.8)	
missing	15 (1.5)	26 (1.4)	
**Sleep quality at T1**			<0.001
Good	214 (21.2)	502 (25.7)	
Normal	774 (76.6)	1352 (69.3)	
Poor	22 (2.2)	94 (4.8)	
missing	1 (0.1)	3 (0.2)	
**Season at 25(OH)D measurement**			<0.001
summer or autumn	575 (56.9)	726 (37.2)	
winter or spring	436 (43.1)	1225 (62.8)	
**Iron supplementary at T1**			0.915
No	859 (85.0)	1662 (85.2)	
Yes	152 (15.0)	289 (14.8)	
**Iron supplementary at T1 and T2 ^†^**			0.001
Never	131 (37.2)	382 (47.5)	
At least one trimester	221 (62.8)	422 (52.5)	
**Iron supplementary at T1, T2 an T3 ^‡^**			0.232
Never	58 (29.1)	170 (34.2)	
At least one trimester	141 (70.9)	327 (65.8)	
	Mean ± SD		
**Age**, year	29.16 ± 3.78	28.39 ± 3.56	<0.001
**Pre-pregnancy BMI**, kg/m^2^	21.33 ± 2.83	21.00 ± 2.80	0.003
**Plasma 25(OH)D at T1**, ng/L	27.50 ± 7.17	13.38 ± 3.87	<0.001
**Gestational age at 25(OH)D measurement**, week	11.53 ± 0.89	11.38 ± 0.96	<0.001
**Weight gain before HB1**, kg	1.30 ± 1.62	1.32 ± 1.84	0.794
**Weight gain before HB2**, kg	3.35 ± 2.07	3.41 ± 2.12	0.503
**Weight gain before HB3**, kg	6.08 ± 2.46	6.35 ± 2.65	0.012
**Weight gain before HB4**, kg	8.27 ± 2.86	8.55 ± 3.13	0.044
**Weight gain before HB5**, kg	10.43 ± 3.34	10.73 ± 3.49	0.052
**Weight gain before HB6**, kg	12.17 ± 3.58	12.60 ± 3.64	0.024
**HB at T1**, g/L	125.96 ± 8.83	125.30 ± 9.06	0.059

HB, hemoglobin; VD, Vitamin D; T1, first trimester; GA, gestational age; HB1, HB of GA from 14 to 17; HB2, HB of GA from 18 to 22; HB3, HB of GA from 23 to 27; HB4, HB of GA from 23 to 27; HB5, HB of GA from 28 to 31; HB6, HB of GA from 36 to 42; † *n* = 1156; ‡ *n* = 696; *n* of HB1 = 1156; *n* of HB2 = 1058; *n* of HB3 = 1098; *n* of HB4 = 696; *n* of HB5 = 658; *n* of HB6 = 555.

**Table 2:** The association * between plasma 25(OH)D in different trimesters and hemoglobin during pregnancy after the corresponding 25(OH)D measurement.

Variable	Observation Number	Model 1		Model 2		Model 3	
		β (se)	*p*	β (se)	*p*	β (se)	*p*
		**Models without interaction**					
25(OH)D in T1 ‡, ng/mL	4419	0.12(0.03)	0.0002	0.08(0.03)	0.0017	0.06(0.03)	0.0177
25(OH)D in T2 ‡, ng/mL	1053	0.17(0.04)	<0.0001	0.16(0.03)	<0.0001	0.15(0.03)	<0.0001
25(OH)D in T3 ‡, ng/mL	337	0.17(0.05)	0.0004	0.12(0.03)	0.0002	0.12(0.03)	0.0006
		**Models with interaction**					
25(OH)D in T1 ‡, ng/mL	4419	−0.01(0.08)	0.9017	−0.05(0.07)	0.4391	−0.07(0.07)	0.2775
GA at Hb measurement, week		−0.23(0.05)	<0.0001	−0.18(0.06)	0.0043	−0.19(0.06)	0.0027
Interaction †		0.005(0.003)	0.0696	0.005(0.003)	0.0464	0.005(0.003)	0.0421
25(OH)D in T2 ‡, ng/mL	1053	−0.05(0.18)	0.7862	−0.07(0.18)	0.7069	−0.07(0.18)	0.6904
GA at Hb measurement, week		0.11(0.15)	0.4503	0.24(0.16)	0.1276	0.25(0.16)	0.1151
Interaction†		0.01(0.01)	0.2314	0.01(0.01)	0.1988	0.01(0.01)	0.2076
25(OH)D in T3 ‡, ng/mL	337	−0.24(0.31)	0.4366	−0.65(0.50)	0.1914	−1.11(1.05)	0.2929
GA at Hb measurement, week		−0.27(0.46)	0.5543	−0.70(0.72)	0.3310	−1.31(1.58)	0.4093
Interaction †		0.01(0.01)	0.1635	0.02(0.01)	0.1122	0.03(0.03)	0.2405

HB, hemoglobin; T1, first trimester; T2, second trimester; T3, third trimester; GA, gestational age. * Analyzed by GEE model; ‡ T1, T2, and T3 were in different models, respectively. † Interaction between VD in each trimester and GA at Hb measurement, respectively. Model 1 was adjusted for GA at HB measurement. Model 2 was adjusted for gestational age at HB measurement, age, gestational age at 25(OH)D measurement, gravidity, parity, season at 25(OH)D measurement, pre-pregnancy BMI, smoking, drinking and tea before pregnancy, sleep quality and physical frequency at trimester of 25(OH)D measurement, weight gain, and corresponding baseline Hb. Model 3 was further adjusted for iron supplementation during pregnancy.

**Table 3:** The association * between 25(OH)D in different trimester and hemoglobin during pregnancy after corresponding 25(OH)D measurement, stratified by whether iron was supplementary during pregnancy or not.

Variable	Iron Supplementary during Pregnancy				*p* for Interaction
	No		Yes		
	β (se)	*p*	β (se)	*p*	
**T1** ^‡^	observation number = 1479		observation number = 2940		
25(OH)D, ng/mL	0.05(0.05)	0.3587	0.09(0.03)	0.0016	0.3140
**T2** ^‡^	observation number = 314		observation number = 759		
25(OH)D, ng/mL	0.09(0.07)	0.2361	0.17(0.03)	<0.0001	0.6991
**T3** ^‡^	observation number = 111		observation number = 226		
25(OH)D, ng/mL	0.15(0.05)	0.0046	0.08(0.04)	0.0486	0.30732

HB, hemoglobin; T1, first trimester; T2, second trimester; T3, third trimester; GA, gestational age. * Analyzed by GEE model; ‡ T1, T2, and T3 were in different models, respectively. Model was adjusted for gestational age at HB measurement, age, gestational age at 25(OH)D measurement, gravidity, parity, season at 25(OH)D measurement, pre-pregnancy BMI, smoking, drinking and tea before pregnancy, sleep quality, and physical frequency at trimester of 25(OH)D measurement, weight gain, and corresponding baseline Hb.

**Table 4:** The association * between VD deficiency in different trimesters and anemia during pregnancy after the corresponding 25(OH)D measurement.

VD Deficiency	Anemia after VD Measurement, Observation Number (%)		Model 1		Model 2		Model 3	
	No	Yes	OR (95% CI)	*p*	OR (95% CI)	*p*	OR (95% CI)	*p*
**T1** ^‡^								
No	1097 (84.2)	206 (15.8)	ref.	-	ref.	-	ref.	-
Yes	2452 (78.7)	664 (21.3)	1.54 (1.16–2.04)	0.0025	1.54 (1.16–2.04)	0.0025	1.42 (1.07–1.88)	0.0167
**T2** ^‡^								
No	398 (75.8)	127 (24.2)	ref.	-	ref.	-	ref.	-
Yes	328 (62.1)	200 (37.9)	1.97 (1.38–2.80)	0.0002	1.93 (1.31–2.85)	0.0010	1.94 (1.30–2.89)	0.0011
**T3** ^‡^								
No	157 (82.2)	34 (17.8)	ref.	-	ref.	-	ref.	-
Yes	103 (70.5)	43 (29.5)	1.13 (1.03–1.24)	0.0109	1.05 (0.96–1.14)	0.2650	1.06 (0.97–1.15)	0.2123

HB, hemoglobin; VD, Vitamin D; T1, first trimester; T2, second trimester; T3, third trimester; GA, gestational age. * Analyzed by GEE model; ‡ T1, T2, and T3 were in different models, respectively. Model 1 was adjusted for GA at HB measurement. Model 2 was adjusted for gestational age at HB measurement, age, gestational age at 25(OH)D measurement, gravidity, parity, season at 25(OH)D measurement, pre-pregnancy BMI, smoking, drinking and tea before pregnancy, sleep quality and physical frequency at trimester of 25(OH)D measurement, weight gain, and corresponding baseline Hb. Model 3 was further adjusted for iron supplementation during pregnancy.

**Table 5:** The association * between VD deficiency in different trimesters and anemia during pregnancy after the corresponding 25(OH)D measurement, stratified by whether iron was supplementary during pregnancy or not.

VD Deficiency	Iron Supplementary during Pregnancy			
	No		Yes	
	OR (95% CI)	*p*	OR (95% CI)	*p*
**T1** ^‡^				
No	ref.	-	ref.	-
Yes	1.22 (0.70–2.12)	0.4765	1.68 (1.20–2.34)	0.0024
**T2** ^‡^				
No	ref.	-	ref.	-
Yes	1.14 (0.58–2.25)	0.6981	2.02 (1.27–3.22)	0.0032
**T3** ^‡^				
No	ref.	-	ref.	-
Yes	1.44 (0.38–5.47)	0.5917	0.84 (0.23–3.09)	0.7948

HB, hemoglobin; VD, Vitamin D; T1, first trimester; T2, second trimester; T3, third trimester; GA, gestational age. * Analyzed by GEE model; ‡ T1, T2, and T3 were in different models, respectively. Model was adjusted for gestational age at HB measurement, age, gestational age at 25(OH)D measurement, gravidity, parity, season at 25(OH)D measurement, pre-pregnancy BMI, smoking, drinking and tea before pregnancy, sleep quality, and physical activity frequency at trimester of 25(OH)D measurement, weight gain, and corresponding baseline Hb.

**Table 6:** The association between 25(OH)D in the first trimester and hemoglobin in different gestational ages.

Hemoglobin, g/L	*n*	Model 1		Model 2		Model 3		Model 4	
		β (se)	*p*	β (se)	*p*	β (se)	*p*	β (se)	*p*
HB at T1 *	2962	0.17 (0.05)	0.0002	0.06 (0.02)	0.0023	0.06 (0.02)	0.0021	0.06 (0.02)	0.0021
HB of GA from 14 to 17 ^†^	1156	0.02 (0.03)	0.5170	−0.01 (0.02)	0.6885	−0.01 (0.02)	0.5556	−0.01 (0.02)	0.5556
HB of GA from 18 to 22 ^†^	1058	0.06 (0.03)	0.0651	0.04 (0.02)	0.0604	0.04 (0.02)	0.0722	0.02 (0.03)	0.3772
HB of GA from 23 to 27 ^†^	1098	0.10 (0.03)	0.0008	0.04 (0.02)	0.0704	0.04 (0.02)	0.1293	0.04 (0.03)	0.1201
HB of GA from 28 to 31 ^‡^	696	0.14 (0.04)	0.0005	0.07 (0.03)	0.0158	0.06 (0.03)	0.0622	0.07 (0.04)	0.0627
HB of GA from 32 to 35 ^‡^	658	0.15 (0.04)	0.0004	0.06 (0.03)	0.0675	0.05 (0.03)	0.1273	0.11 (0.04)	0.0105
HB of GA from 36 to 42 ^‡^	555	0.17 (0.35)	<0.0001	0.08 (0.03)	0.0245	0.06 (0.03)	0.0916	0.14 (0.05)	0.0036

HB, hemoglobin; T1, first trimester; T2, second trimester; T3, third trimester; GA, gestational age. Model 1 was adjusted for GA at HB measurement. Model 2 was adjusted for age, gestational age at 25(OH)D measurement, gravidity, parity, season at 25(OH)D measurement, pre-pregnancy BMI, smoking, drinking, and tea before pregnancy, sleep quality and physical activity frequency at T1, gestational age and weight gain at the corresponding gestational age of HB measurement and baseline Hb. Model 3 * was further adjusted for iron Supplementary at T1; Model 3 † was further adjusted for iron Supplementary from T1 to T2; Model 3 ‡ was further adjusted for iron Supplementary from T1 to T3. Model 4 was adjusted for the variables in Model 3, but baseline Hb was Hb in T1.

**Table 7:** The association between plasma 25(OH)D change and hemoglobin change between different trimesters.

25(OH)D Change, ng/mL	*n*	Crude Model		Model 2		Model 3	
		β (se)	*p*	β (se)	*p*	β (se)	*p*
**From T1 to T2**	**HB change from T1 to T2** ^†^, g/L						
	516	−0.09 (0.03)	0.0013	−0.03 (0.03)	0.3813	−0.00 (0.03)	0.9225
	**HB change from T2 to T3** ^‡^, g/L						
	273	0.15 (0.06)	0.0070	0.15 (0.05)	0.0040	0.11 (0.05)	0.0430
**From T2 to T3**	**HB change from T2 to T3** ^‡^, g/L						
	216	0.05 (0.06)	0.4253	0.15 (0.06)	0.0153	0.13 (0.06)	0.0312
**From T1 to T3**	**HB change from T1 to T3** ^‡^, g/L						
	293	0.00 (0.05)	0.9189	0.17 (0.05)	0.0008	0.15 (0.05)	0.0027

HB, hemoglobin; T1, first trimester; T2, second trimester; T3, third trimester; GA, gestational age. Model 2 was adjusted for age, gestational age at later 25(OH)D measurement, gravidity, parity, season at later 25(OH)D measurement, pre-pregnancy BMI, smoking, drinking, and tea before pregnancy, sleep quality and physical activity frequency at later GA, change of gestational age and weight gain at the corresponding gestational age of HB measurement, corresponding baseline HB and 25(OH)D. Model 3 † was further adjusted for iron Supplementary from T1 to T2; Model 3 ‡ was further adjusted for iron supplementation from T1 to T3.

## Data Availability

The data presented in this study are available on request from the corresponding author. The data are not publicly available because they contain information that could compromise the privacy of research participants.

## Supplementary Materials

The following supporting information can be downloaded at: https://www.mdpi.com/article/10.3390/nu14122455/s1, Supplementary Table S1. The association between 25(OH)D in the second and third trimester and hemoglobin in different gestational ages, respectively; Supplementary Table S2. The association between 25(OH)D in different trimesters and hemoglobin in different gestational ages were stratified by whether iron was supplementary during pregnancy or not.
