# Supplementary material for: Association of Vitamin D in Different Trimester with Hemoglobin during Pregnancy
Source: Nutrients. 2022 Jun 14;14(12):2455. doi: 10.3390/nu14122455 (PMC9230968; doi:10.3390/nu14122455)
Supplement: Supplementary file 1 [file nutrients-14-02455-s001.zip › nutrients-1756076-supplementary.pdf]

Supplementary Table S1. The association between 25(OH)D in second and third trimester and hemoglobin in different gestational age, respectively.

Hemoglobin, g/L	N	Model 1		Model 2		Model 3		Model 4	
		β(se)	*P*	β(se)	*P*	β(se)	*P*	β(se)	*P*
25(OH)D in T2									
HB of GA from 23 to 27†	516	0.06 (0.03)	0.0790	0.05 (0.03)	**0.0400**	0.06 (0.03)	**0.0131**	0.06 (0.03)	**0.0131**
HB of GA from 28 to 31‡	320	0.14 (0.04)	**0.0007**	0.11 (0.03)	**0.0011**	0.11 (0.04)	**0.0027**	0.13 (0.04)	**0.0021**
HB of GA from 32 to 35‡	307	0.18 (0.04)	**<0.0001**	0.06 (0.03)	0.0772	0.05 (0.04)	0.1462	0.12 (0.05)	**0.0081**
HB of GA from 36 to 42‡	265	0.18 (0.05)	**0.0002**	0.08 (0.04)	**0.0277**	0.07 (0.04)	0.0654	0.11 (0.06)	**0.0658**
25(OH)D in T3									
HB of GA from 32 to 35	293	0.12 (0.04)	**0.0036**	0.07 (0.03)	**0.0185**	0.06 (0.03)	**0.0416**	0.06 (0.03)	**0.0416**
HB of GA from 36 to 42	247	0.17 (0.05)	**0.0002**	0.11 (0.04)	**0.0018**	0.11 (0.04)	**0.0039**	0.12 (0.04)	**0.0058**

HB, hemoglobin; T1, first trimester; T2, second trimester; T3, third trimester; GA, gestational age.

Model 1 was adjusted for GA at HB measurement.

Model 2 was adjusted for age, gestational age at 25(OH)D measurement, gravity, parity, season at 25(OH)D measurement, pre-pregnancy BMI, smoking, drink and tea before pregnancy, sleep quality and physical frequency at GA of 25(OH)D measurement, gestational age and weight gain at the corresponding gestational age of HB measurement and baseline Hb.

Model 3† was further adjusted for iron Supplementary from T1 to T2; Model 3‡ was further adjusted for iron Supplementary from T1 to T3.

Model 4 was adjusted for the variables in Model 3 but baseline Hb was HB of corresponding GA before and closest to 25(OH)D measurement.

Supplementary Table S2. The association between 25(OH)D in different trimesters and hemoglobin in different gestational age stratified by whether iron supplementary during pregnancy or not.

Hemoglobin, g/L	Iron supplementary during pregnancy before Hb measurement				*P* for interaction
	No		Yes		
	β(se)	*P*	β(se)	*P*	
25(OH)D in T1					
HB at T1	0.04 (0.03)	0.1800	0.09 (0.03)	0.0034	0.1996
HB of GA from 14 to 17	0.00 (0.04)	0.9133	-0.01 (0.03)	0.7606	0.9190
HB of GA from 18 to 22	0.03 (0.04)	0.4713	0.06 (0.03)	0.0293	0.3681
HB of GA from 23 to 27	0.00 (0.04)	0.9009	0.07 (0.03)	0.0177	0.3685
HB of GA from 28 to 31	0.00 (0.06)	0.9970	0.10 (0.03)	0.0045	0.3970
HB of GA from 32 to 35	0.01 (0.06)	0.8476	0.06 (0.04)	0.0744	0.4019
HB of GA from 36 to 42	-0.01 (0.08)	0.8979	0.09 (0.04)	0.0251	0.4130
25(OH)D in T2					
HB of GA from 23 to 27	0.03 (0.05)	0.5870	0.09 (0.03)	**0.0033**	0.2360
HB of GA from 28 to 31	0.01 (0.08)	0.8832	0.12 (0.04)	**0.0015**	0.9161
HB of GA from 32 to 35	0.07 (0.08)	0.3370	0.05 (0.04)	0.2457	0.5099
HB of GA from 36 to 42	0.06 (0.10)	0.5163	0.07 (0.04)	0.0757	0.8900
25(OH)D in T3					
HB of GA from 32 to 35	0.07 (0.05)	0.2204	0.08 (0.04)	0.0566	0.1000
HB of GA from 36 to 42	0.12 (0.07)	0.1045	0.15 (0.04)	**0.0003**	0.6040

HB, hemoglobin; T, first trimester; T2, second trimester; T3, third trimester; GA, gestational age.
Model was adjusted for age, gestational age at 25(OH)D measurement, gravity, parity, season at 25(OH)D measurement, pre-pregnancy BMI, smoking, drink and tea before pregnancy, sleep quality and physical frequency at GA of 25(OH)D measurement, gestational age and weight gain at the corresponding gestational age of HB measurement and baseline Hb.
